# An improved procedure for isolation of high-quality RNA from nematode-infected *Arabidopsis* roots through laser capture microdissection

**DOI:** 10.1186/s13007-016-0123-9

**Published:** 2016-04-26

**Authors:** Muhammad Shahzad Anjam, Yvonne Ludwig, Frank Hochholdinger, Chisato Miyaura, Masaki Inada, Shahid Siddique, Florian M. W. Grundler

**Affiliations:** INRES - Molecular Phytomedicine, Rheinische Friedrich-Wilhelms-Universitaet Bonn, Karlrobert-Kreiten-Straße 13, 53115 Bonn, Germany; INRES - Crop Functional Genomics, Rheinische Friedrich-Wilhelms-Universitaet Bonn, Friedrich-Ebert-Allee 144, 53113 Bonn, Germany; Department of Biotechnology and Life Science, and Global Innovation Research Organization, Tokyo University of Agriculture and Technology, 2-24-16 Nakacho, Koganei, Tokyo 184-8588 Japan; Institute of Molecular Biology and Bio-technology, Bahauddin Zakariya University, Multan, Pakistan

**Keywords:** Syncytium, LCM, Root, Arabidopsis, RNA degradation, Cyst nematode, Nematode

## Abstract

**Background:**

Cyst nematodes are biotrophs that form specialized feeding structures in the roots of host plants, which consist of a syncytial fusion of hypertrophied cells. The formation of syncytium is accompanied by profound transcriptional changes and active metabolism in infected tissues. The challenge in gene expression studies for syncytium has always been the isolation of pure syncytial material and subsequent extraction of intact RNA. Root fragments containing syncytium had been used for microarray analyses. However, the inclusion of neighbouring cells dilutes the syncytium-specific mRNA population. Micro-sectioning coupled with laser capture microdissection (LCM) offers an opportunity for the isolation of feeding sites from heterogeneous cell populations. But recovery of intact RNA from syncytium dissected by LCM is complicated due to extended steps of fixation, tissue preparation, embedding and sectioning.

**Results:**

In the present study, we have optimized the procedure of sample preparation for LCM to isolate high quality of RNA from cyst nematode induced syncytia in Arabidopsis roots which can be used for transcriptomic studies. We investigated the effect of various sucrose concentrations as cryoprotectant on RNA quality and morphology of syncytial sections. We also compared various types of microscopic slides for strong adherence of sections while removing embedding material.

**Conclusion:**

The use of optimal sucrose concentrations as cryoprotection plays a key role in RNA stability and morphology of sections. Treatment with higher sucrose concentrations minimizes the risk of RNA degradation, whereas longer incubation times help maintaining the morphology of tissue sections. Our method allows isolating high-quality RNA from nematode feeding sites that is suitable for downstream applications such as microarray experiments.

## Background

Plant-parasitic nematodes (PPNs) are obligate biotrophs that cause significant damage to almost every economically important crop [27]. PPNs are classified based on their feeding habits as either ectoparasites or endoparasites. The endoparasitic root-knot (*Meloidogyne* spp.) and cyst nematodes (*Globodera* spp. and *Heterodera* spp.) are sedentary parasites of roots and the primary nematode pathogen of a wide range of crops. Infective stage juveniles (J2s) of cyst nematodes invade the host root and migrate intracellularly until they reach the vascular cylinder. There, these nematodes select an initial syncytial cell (ISC) and become sedentary. Within 24 h of the ISC selection the cells adjacent to the ISC appear hypertrophied and fused together due to local dissolution of the cell walls [32]. Three days after ISC selection, the cells that have been incorporated into the syncytium are enlarged and exhibit the features of a typical syncytial cell such as condensed cytoplasm and enrichment of ribosomes, endoplasmic reticulum, mitochondria and plastids. The nuclei have become enlarged, and several smaller vacuoles are present in the cytoplasm. Moreover the outer cell walls thicken to withstand the high osmotic pressure within the syncytium [5]. The syncytium serves as the sole source of nutrients and water for the rest of the nematode’s successive growth and development. A cocktail of secretions, which are synthesised in the oesophageal glands of the nematodes, are responsible for the modulation of the plant’s defence and the developmental pathways that lead to the formation of a syncytium [8, 9, 12, 29]. Therefore understanding the function of host genes that are required for syncytium development is crucial for identifying novel targets for nematode resistance.

Compatible interaction is best studied between cyst nematode *Heterodera schachtii* and the model plant *Arabidopsis thaliana*. In recent years a lot of work with this particular pathosystem has shown that the development of the syncytium is accompanied by massive transcriptomic, metabolomic and proteomic changes [11, 13, 31]. Nevertheless isolating pure syncytial material and extracting good quality RNA from Arabidopsis roots for downstream application, such as microarray analyses, qPCR and RNA-seq is challenging. Puthoff et al. [25] used the entire root systems of cyst nematode-infected Arabidopsis plants to perform the first transcriptomic studies. However including non-infected cells dilutes the feeding cell specific mRNA expression profile [10, 31]. In a step forward, Szakasits et al. [31] tried to overcome this limitation by collecting the pure cytoplasm of feeding cells through micro-aspiration. But producing a sufficient amount of material for analysis using this technique is laborious. Moreover it works better on larger, well-developed syncytial cells as compared to syncytial tissues during the early stages of development. An alternative and promising technique for collecting pure cells is laser capture microdissection (LCM).

LCM is becoming a popular tool among cell biologists because it allows isolation of RNA, DNA, proteins and metabolites from a heterogeneous cell population. Current LCM techniques are based on two principles. In the “touch-free” approach, cells identified under a microscope are excised by a laser beam and removed from their tissue context by gravity or by catapulting them away with a photonic pulse. Alternatively, cells are fused to a plastic membrane and are subsequently removed mechanically from the remaining tissue (reviewed in Ludwig and Hochholdinger [22]).

Successful LCM requires good morphological preservations of tissues. Furthermore, isolated nucleic acids (RNA or DNA), proteins or metabolites must be of high quality to ensure their utility for downstream applications. Therefore, tissue preparation is the crucial step towards successful RNA isolation of samples generated by LCM. Since LCM requires micro-sections of 2–20 µm, samples must be fixed and embedded prior to sectioning and mounting them on the slides. During this multistep procedure, RNA integrity remains at high risk of degradation due to the presence of RNases. Samples for immuno-histological analyses are generally fixed in paraformaldehyde and embedded in paraffin to ensure well-preserved tissue morphology. In contrast, ethanol:acetic acid (EAA) solution is preferred for fixation in experiments involving RNA extraction because paraformaldehyde cross links with RNA and thus makes its recovery difficult [14, 17, 18, 30]. After fixation the tissues are embedded either in paraffin at room temperature or in an optimum cutting temperature (OCT) medium at −20 °C (cryosectioning). While OCT-embedded samples are sectioned through a cryomicrotome at −20 °C, the paraffin-embedded samples are sectioned at room temperature. Cryosectioning is often preferred over sectioning of paraffin-embedded samples because processing the tissue at a low temperature reduces the activity of RNases and increase yield [1, 23]. Nevertheless keeping the RNA intact during the various steps of the cryosectioning for LCM poses a challenge and entails fixing and processing tissues. For example, plant cells contain big vacuoles for water storage, which may form crystals because of fast freezing of samples during cryosectioning leading to the destruction of cellular structures. Similarly, it is challenging to maintain the histological preservation during cryosectioning so that the target cells can be distinguished from others cell types. Therefore a number of tissue fixation and embedding procedures have been developed to ensure optimised handling of tissues with different physical rigidity [4].

Nakazono et al. [23] were the first to report application of LCM in plant samples by isolating the epidermis and vascular tissue cells of maize plants, which were subsequently used in microarray studies. Soon after, Kerk et al. [17] presented an RNA isolation technique using the paraffin-embedded sections of a variety of plant tissues. Further the LCM procedure has been applied to isolate syncytial material from soybean roots infected with cyst nematode *H. glycines* [15, 18, 19]. These authors applied paraffin embedding to process the syncytial samples and showed that a number of genes are differentially regulated in these plants upon nematode infection. But the utility of this protocol for nematode-infected Arabidopsis roots remains questionable as it may lead to poor quality RNA extraction. LCM was also used to capture giant cells and isolate RNA induced by root-knot nematodes in Arabidopsis, rice and tomato during different stages of infection [1, 3, 7, 16, 24, 26]. However no protocol describing the isolation of syncytial cells from Arabidopsis have been described so far. In the present study we optimised the LCM procedure for the isolation of syncytial cells from Arabidopsis roots in such a manner that their morphology can be preserved and high-quality RNA can be isolated for downstream applications such as microarray analyses.

## Results

To assess the potential of a previously described giant cell RNA isolation protocol in Arabidopsis for its utility for syncytial samples, we grew Arabidopsis plants in KNOP medium and infected them with J2 nematodes of *H. schachtii*. Root segments containing syncytia were cut at 5 days post inoculation (dpi). The infected root segments were subsequently fixed in EAA (3:1) and processed for LCM as described previously [1, 23, 24]. However, RNA was heavily degraded in these samples and was unusable for downstream applications (data not shown).

**Fig. 1:**
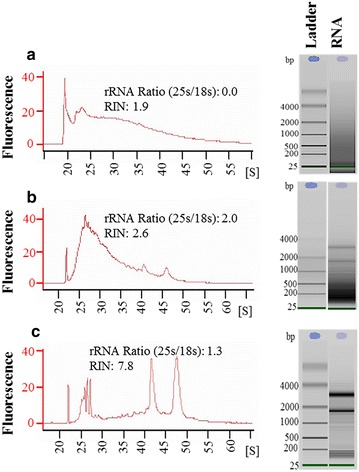
Influence of tissue fixation and embedding on RNA quality. Electropherograms indicating the quality of RNA extracted from Arabidopsis root segments infected with nematodes at 5 dpi. **a** RNA was extracted from tissues following all steps of fixation, cryoprotection and embedding, however before LCM. Number of root pieces (containing syncytium) used for extraction: 10; RNA concentration: 3407 pg/μL. **b** Tissue samples after fixation and cryoprotection but before embedding. There is a splice between Ladder and RNA samples; however, both runs are from the same chip and were put together to facilitate visualization. Number of root pieces (containing syncytium) used for extraction: 15; RNA concentration: 4693 pg/μL. **c** Control samples with fixation but without cryoprotection or embedding. Number of root pieces (containing syncytium) used for extraction: approx. 25 RNA concentration: 1440 pg/μL. **a** The experiment was performed once. **b**, **c** The experiments were repeated three times and data from one representative experiment is provided. *S* seconds, *bp* base pair

To identify the crucial step in which the RNA was degraded, we hand-dissected root segments containing syncytia at 5 dpi and isolated RNA at three different steps (Table 1). The subsequent quality analysis revealed that the RNA was already degraded before embedding in OCT. Hence sections mounted on the slides already contained deteriorated RNA (Fig. 1a, b). Conversely the RNA isolated from syncytial tissues that were fixed but not treated for cryoprotection produced high-quality RNA (Fig. 1c). We concluded that the cryoprotection procedure needs to be modified to obtain good quality RNA.

**Table 1:** An overview of influence of tissue fixation and embedding on RNA quality

Method	Fixation	Cryoprotection in sucrose	Embedding	RNA quality
A	Yes	Yes	Yes (OCT)	Degraded
B	Yes	Yes	No	Degraded
C	Yes	No	No	Good

A, B and C refers to three different steps from which RNA was isolated. (A) RNA was extracted from tissues following all steps of fixation, cryoprotection and embedding, however before LCM. (B) Tissue samples after fixation and cryoprotection but before embedding. (C) Control samples with fixation but without cryoprotection or embedding

### Modification of cryoprotection steps yielded good quality RNA

**Fig. 2:**
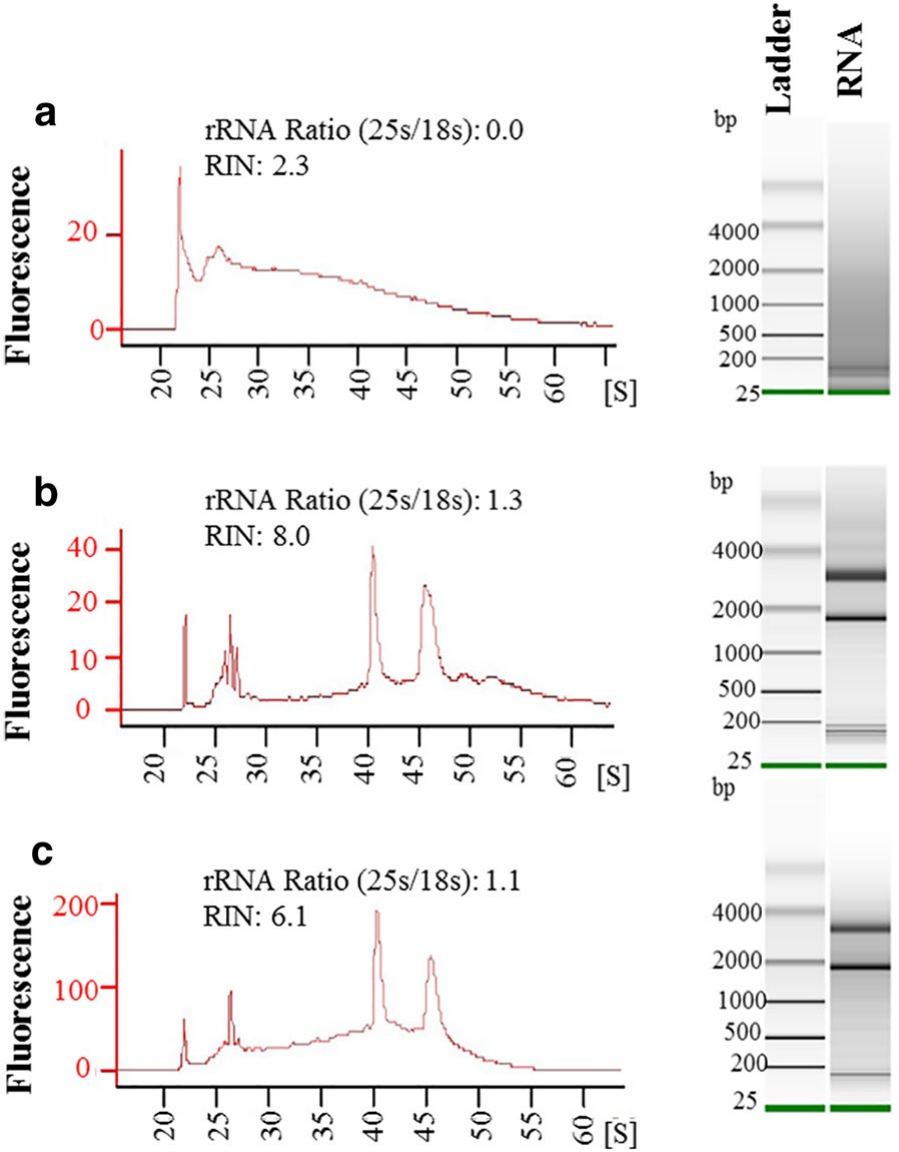
Quality assessment of RNA isolation from samples after modifying the tissue preparation steps. **a** RNA isolated after direct incubation in 15 % sucrose solution (low sucrose concentration treated samples). Number of root pieces (containing syncytium) used for extraction: 10; RNA Concentration: 2514 pg/μL. **b** RNA isolated after direct incubation in 34 % sucrose (high sucrose concentration treated samples). Number of root pieces (containing syncytium) used for extraction: 10; RNA concentration: 2253 pg/μL. **c** RNA isolation from samples B after LCM. Number of syncytium used for cryosectioning: 30; RNA concentration: 6297 pg/μL. **a**–**c** The experiments were repeated three times and data from one representative experiment is provided. *S* seconds, *bp* base pair

To investigate whether RNA degradation might have occurred because of multiple incubations in different sucrose concentrations (10 % for 3 h, 15 % for 3 h and 34 % overnight) during cryoprotection; we modified the tissue preparation procedure by reducing the sucrose incubation steps. For this, root segments containing infected tissues were cut and fixed in EAA solution followed by a direct incubation in 15 % sucrose solution for 3 h (low concentration sucrose-treated samples). These samples were then left overnight in 34 % sucrose solution. Alternatively fixed tissues were directly incubated in 34 % for 5 h (high concentration sucrose-treated samples). Subsequently, tissues from low- and high-concentration sucrose-treated samples were embedded in OCT, cryosectioned and used for RNA isolation. While the RNA from low-concentration sucrose-treated tissues was heavily degraded (Fig. 2a), the sections from high-concentration sucrose-treated tissues showed high-quality RNA (Fig. 2b). Therefore these samples were further processed (mounting on slides, and removing OCT) to isolate syncytial sections through LCM. Subsequent RNA isolation showed a slight degradation in quality of RNA; however, both the quantity and quality of RNA was still reasonable to perform downstream applications (Fig. 2c). We concluded that incubating fixed samples directly in a 34 % sucrose solution for 5 h improves the RNA quality considerably.

### Incubation in higher sucrose concentrations affected tissue morphology

**Fig. 3:**
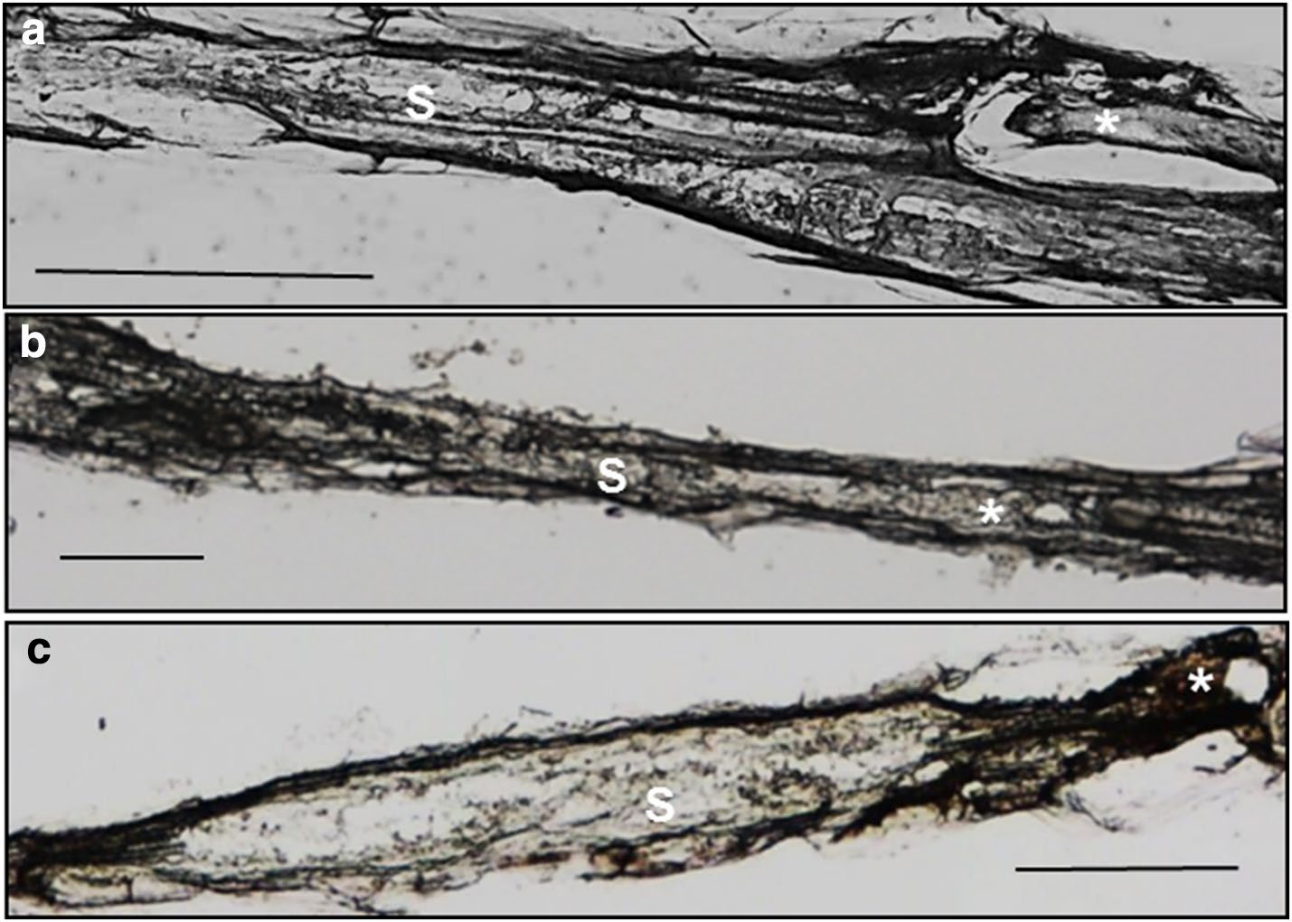
Morphology of longitudinal syncytial samples (10 µm thin)upon different sucrose treatment. **a** Infected root segments upon direct incubation in 15 % sucrose solution (low sucrose concentration treated samples). **b** Infected root segments upon direct incubation in 34 % sucrose (high sucrose concentration treated samples). **c** Infected root segments upon direct overnight incubation in 34 % sucrose. *Asterisk* (*) nematode, *S* syncytium, *scale bar* 100 µM

Although incubating fixed samples directly in 34 % sucrose for 5 h resulted in improved RNA quality, the tissue morphology was poor (Fig. 3a, b). We reasoned that this might be due to shorter and direct incubation in high concentration sucrose, which may lead to sudden loss of tissue pressure. Therefore we increased the vacuum infiltration of samples in 34 % sucrose from 15 to 45 min followed by an overnight incubation at 4 °C. As a consequence, tissue morphology was improved to normal (Fig. 3c). The RNA quality of the samples was not affected by increasing the incubation time.

### Poly-l-lysine-coated glass slides strongly adhere to micro-sections

Different types of glass slides with diverse features are available. In this study, four types of slides were tested to obtain a strong tissue adherence: (a) polyethylene teraphthalate (PET)-membrane-coated slides, (b) polyethylene napthalate (PEN)-membrane-coated slides, (c) Superfrost^®^ positively charged slides, and (d) poly-l-lysine-coated glass slides. We mounted 10 µm sections on PET- and PEN-membrane-coated glass slides and washed them with PBS buffer and 70 % ethanol to remove the OCT medium. The complete removal of the OCT medium is necessary for laser cutting. However during washing, all sections were washed away from the slides. We pre-treated the slides with UV light for 30 min to 4 h to enhance adherence, but the majority of the sections was still washed off during the washing step. Next we used Superfrost^®^ positively-charged glass slides, on which tissue retention was greatly improved, while the use of poly-l-lysine-coated glass slides proved robust in holding the tissues during the washing step. Hence, in all subsequent experiments, we used poly-l-lysine-coated glass slides, and the laser capture machine was also calibrated to glass slides.

### In vitro amplification of cDNA

**Fig. 4:**
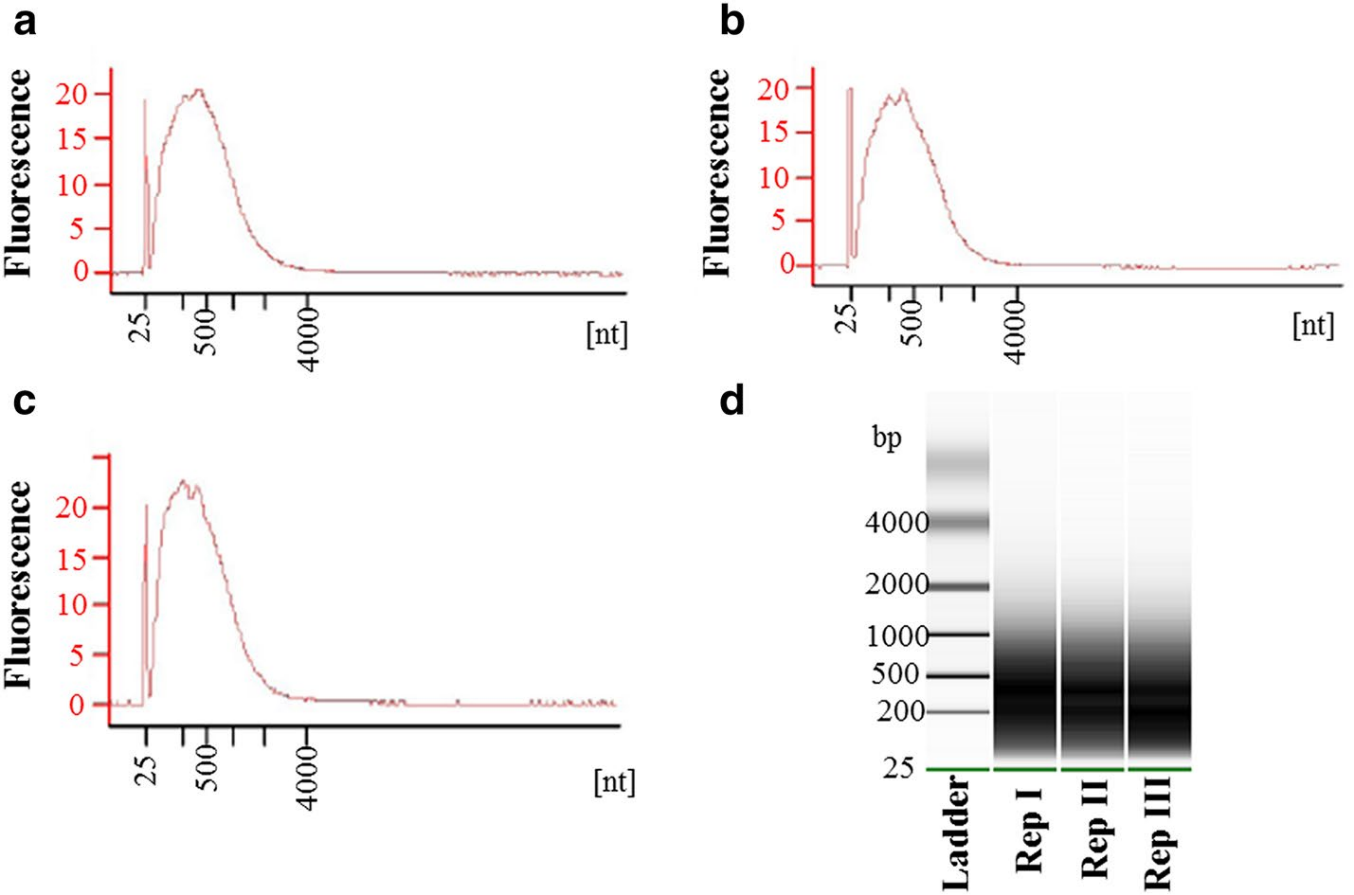
Amplification of cDNA, where **a**–**c** represents three replications of syncytial samples processed after LCM. The virtual gel generated by an Agilent 2100 Bioanalyzer is shown in **d**. *nt* nucelotide, *bp* base pair

**Fig. 5:**
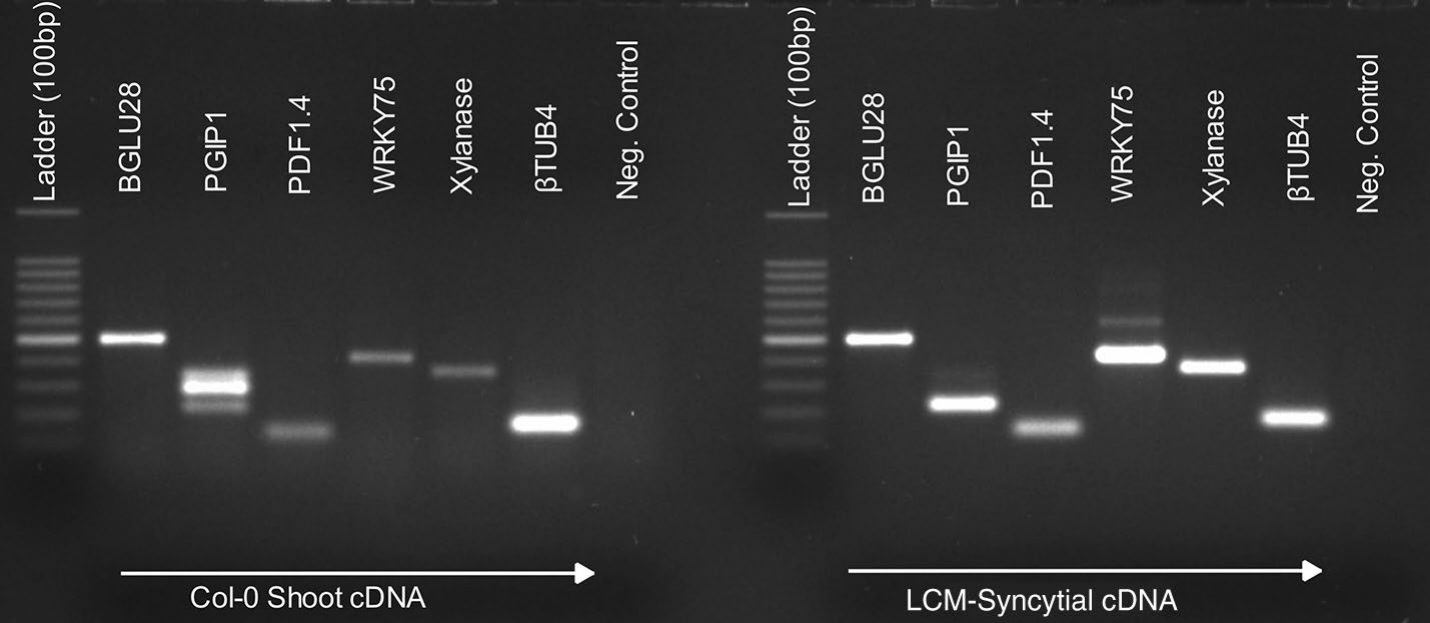
Analysis of gene expression in uninfected leaf tissues and in 5-days-old syncytium by RT-PCR. Negative control is without template

To assess the quality of RNA isolated through our protocol for downstream applications, cDNA was synthesised from 5-days-old syncytial using NuGEN’s Ovation Pico WTA System according to the manufacturers’ instructions. Subsequent analysis showed that enough high quality cDNA was amplified (Fig. 4a–c). The amplified cDNA was used to perform further steps of hybridization with the GeneChip^®^ Arabidopsis ATH1 Genome (Affymetrix UK Ltd). The data analysis showed that amplified cDNA was suitable for microarray experiments (data not shown). Whereas the detailed results from microarray will be presented elsewhere, we analysed the expression of six different genes (BGLU28, PGIP1, PDF1.4, WRKY76, Xylanase, and ß tubulin4) in 5-days-old syncytial samples by RT-PCR (see methods for details). These genes were selected based on intensity of expression in syncytium in our microarray data (data not shown). Under optimized PCR conditions, five out of six genes produced a single band of the expected size from LCM-derived syncytial RNA. However, WRKY76 showed an extra band in syncytial samples suggesting unspecific binding of primers (Fig. 5). Nevertheless, these results confirm that RNA isolated through LCM protocol as described in this manuscript can be used to study gene expression in syncytium during early stages of nematode infection.

## Discussion

After the successful use of LCM on animal and human tissues, it has become increasingly popular among plant scientists because of its ability to isolate a single cell-type from a heterogeneous population. The collected material can be used for a broad range of downstream molecular studies, for example, qPCR, RNAseq, microarray and proteomics. In contrast to animal cells the presence of a cell wall and large vacuole in plants necessitates modifications to preserve cell morphology and macromolecules, such as RNA and proteins. As the use of LCM in plant sciences expands, a variety of optimized protocols for specific cell-type harvest have been established. However in contrast to a typical plant cell with a large central vacuole, the cyst-nematode-induced syncytium is a distinctive structure surrounded by a thick cell wall that contains several smaller vacuoles. Therefore performing LCM on syncytial samples may necessitate certain modifications in existing protocols.

In this study we used a previously described protocol for LCM on giant cells induced by root-knot nematodes in Arabidopsis as a starting point [1]. However when we incubated the samples in various sucrose concentrations, the RNA in the samples was highly degraded (Fig. 1a). In contrast incubating samples directly in a 34 % sucrose solution preserved the RNA integrity, indicating that RNase activity may not completely stop at lower sucrose concentrations. A higher concentration of sugar minimise the availability of water in the tissues, which may reduce enzymatic activities including that of RNase. The cryoprotective use of higher sucrose concentrations (20 or 30 %) has already been reported in animal tissues when performing LCM and immunohistochemistry [6, 20, 21]. The use of an RNase inhibitor, for example, RNase later^®^, maintains high-quality RNA; however RNase later^®^ severely compromised the morphology of the sections in some reports [2].

Although the 34 % sucrose concentration significantly increased RNA quality, the morphology of the sections was strongly compromised. We reasoned that this may be due to direct tissue incubation in a higher concentration sucrose solution (34 %), which may result in a rapid loss of water and thus shrunk tissues. Indeed tissue morphology was maintained through longer vacuum infiltration and an overnight incubation in a 34 % sucrose solution (see “Methods”). The slow intake of solution might have helped the cells maintain their shape.

After the morphology of the sections was satisfying, the embedding medium was removed completely by washing which is a perquisite for the laser beam to function properly. The commercially available OCT medium is water soluble and can be removed easily by washing with PBS buffer. However the root sections can also be washed away unless they adhere strongly to the slides. We tried membrane-covered slides (PET and PEN), which permit very fine dissection of target cells, and the targeted area can be catapulted as a single intact piece, but they proved very poor in holding root sections during the washing step. In contrast, Ramsay et al., [26] used PEN slides for the LCM of giant cells from tomato roots, and Barcala et al. [1] used Superfrost^®^ positively-charged glass slides for giant cells in Arabidopsis roots. However we found that poly-l-lysine-coated glass slides robustly anchored the frozen sections of 5-day-old syncytia.

In conclusion, we have shown that fixation with EAA and a direct infiltration with 34 % sucrose, prior to embedding in OCT medium generates syncytial samples with well-preserved morphology that are suitable to distinguish syncytial cells from uninfected surrounding tissue during LCM. Moreover, high quality RNA was extracted from LCM-processed syncytial samples, which was suitable for downstream applications such as microarrays analysis and RT-PCR. Importantly, we were able to analyse the RNA quality prior to amplification, which has not been shown previously [24]. Our protocol will help analysing gene expression during early stages of cyst nematode infection, which may help understanding genes and pathways that are important for nematode development. In addition, development of analytical tools such as syncytium-specific promoters will be greatly facilitated by application of this methodology.

## Methods

### Tissue preparation and sample collection

10-day-old *Arabidopsis thaliana* plants were grown on KNOP medium and inoculated with 60–70 juveniles of *Heterodera schachtii* as described previously [28]. Subsequently, 5-day-old infected root samples were hand-dissected and vacuum infiltrated in EAA solution (3:1) for 20 min. Afterwards, EAA solution was replaced with fresh solution and samples were incubated at 70 rpm on an undulating shaker at 4 °C for 1 h.

### Cryoprotection

The fixative solution was replaced with a 34 % sucrose solution prepared in 0.01 M PBS buffer (treated with 0.1 % DEPC), pH 7.4. Safranin-O (0.01 % in water; Cat. No. S2255. Sigma-Aldrich, USA) was added to visualise the otherwise transparent tissues. Moreover, to facilitate solution penetration, a vacuum was applied for 45 min at 4 °C. Subsequently, samples were incubated in an undulating shaker at 4 °C for 24 h.

### Embedding

The tissues were horizontally embedded in a tissue freezing medium, OCT (Polyfreeze^®^, Cat No. 25112. Poly Sciences, Germany) using Cryomold Tissue-Tek^®^ (Cat No. E62534. EMS, USA). The embedded tissues were frozen by touching the back of cryomolds to liquid nitrogen. The embedded samples (OCT blocks) were stored at −80 °C until cryosectioning.

### Cryosectioning and laser capture microdissection

The OCT blocks containing tissues were left inside a cryomicrotome (CM 1850 UV, Leica, Germany) pre-cooled to −20 °C for 15 min for acclimatisation before making sections. Longitudinal sections of 10 µm were mounted on poly-l-lysine-coated glass slides (Cat no. J2800AMNZ. Manzel Glaser, Germany). After collecting 10–15 sections on the cryomicrotome platform, a slide at RT was suddenly placed over the sections to adhere them. The slides with sections were stored in 70 % ethanol, which was already pre-chilled in the cryomicrotome.

For OCT removal and dehydration, the slides were treated twice with 70 % ethanol for 30 s each at RT, 95 % ethanol overnight at −20 °C and 99.8 % ethanol for 2 min at RT. After air drying the slides at RT for 2 min, the sections were immediately processed for LCM, and the remaining slides were stored in a box containing silica gel. The LCM captured syncytium cells were collected in RNAse-free adhesive caps (Cat. No. 415190-9211-001 Zeiss, Germany) using the following settings for PALM Microbeam laser capture (Zeiss, Germany). Energy: 55–57, Focus: 79–80, Cutting Program: “CloseCut+AutoLPC” RNA Extraction, Quality Assessment and Gene expression: 10 µl extraction buffer from the PicoPure^®^ RNA isolation kit (Cat no. KIT0204, ThermoFisher Scientific, Germany) was added to an adhesive cap, incubated at 42 °C for 20 min. The extraction mixture was briefly spun down in a table centrifuge at 8000 rpm and was stored at −80 °C until RNA extraction. The later steps of RNA extraction followed the instructions provided in the kit by the manufacturer. The quality of the isolated RNA was tested with an Agilent 2100 Bioanalyzer using the Agilent RNA 6000 Pico kit (Cat. No. 5067-1513. Agilent, Germany). cDNA synthesis was done with NuGEN’s Ovation Pico WTA System (Cat. No. 3302-12. Nugen, USA) according to the manufacturers’ instructions, starting with 1–50 ng of total RNA. NuGEN’s Encore Biotin Module (Cat. No. 4200-12. Nugen, USA) was used to fragment 3.95 µg cDNA followed by Biotin-labelling according to the manufacturers’ instructions. The gene expression was analysed through RT-PCR. Each sample contained 6 μL Master Mix, 1 μL template cDNA (1:20 dilution), 0.5 μL of forward and 0.5 μL of reverse primers (10 μM) and water in 20 μL of total reaction volume. Primers are given in Table 2.

**Table 2:** Primer sequences used in this study

Gene	Primer	Sequence	Product size	Locus
BGLU28	Forward	GCTACGACACTGGCAACAAA	501	AT2G44460
	Reverse	TGTGATTTGTTACTCGCCATTG		
PGIP1	Forward	CCATTCCAAGTTCTCTCTCTACG	221	AT5G06860
	Reverse	AGCATCACCTTGGAGCTTGT		
PDF1.4	Forward	CTTCCTTATAGCTTCCACTGAGAT	130	AT1G19610
	Reverse	AGCACGTTCCCATCTCTTAC		
WRKY75	Forward	ATGGAGGGATATGATAATGGGTC	418	AT5g13080
	Reverse	GCATTTGAGTGAGAATATGCTCG		
Xylanase	Forward	CTGTTCTTGGTCGTCCCATT	360	AT1G10050
	Reverse	CGACAACGAACGTTTTGAGA		
Beta tubulin 4	Yes	TTTCCGTACCCTCAAGCTCG	160	AT5G44340
		GTGAAGCCTTGCGAATGGGA		
